# Paralytic Shellfish Poisoning (PSP) in Mussels from the Eastern Cantabrian Sea: Toxicity, Toxin Profile, and Co-Occurrence with Cyclic Imines

**DOI:** 10.3390/toxins13110761

**Published:** 2021-10-27

**Authors:** Tamara Rodríguez-Cabo, Ángeles Moroño, Fabiola Arévalo, Jorge Correa, Juan Pablo Lamas, Araceli E. Rossignoli, Juan Blanco

**Affiliations:** 1 Instituto Tecnolóxico para o Control do Medio Mariño (Intecmar), Peirao de Vilaxoán s/n, 36611 Vilagarcía de Arousa, Spain; trodriguez@intecmar.gal (T.R.-C.); amoronho@intecmar.gal (Á.M.); farevalo@intecmar.gal (F.A.); jcorrea@intecmar.gal (J.C.); plamas@intecmar.gal (J.P.L.); 2 Centro de Investigacións Mariñas (CIMA), Xunta de Galicia, Pedras de Corón s/n, 36620 Vilanova de Arousa, Spain; araceli.escudeiro.rossignoli@xunta.gal

**Keywords:** shellfish, gymnodimine A, 13-desmethyl spirolide C, Cantabria, Basque Country, *Alexandrium*, LC–FLD, LC–MS/MS, HILIC, mouse bioassay

## Abstract

In the late autumn of 2018 and 2019, some samples taken by the official monitoring systems of Cantabria and the Basque Country were found to be paralytic shellfish poisoning (PSP)-positive using a mouse bioassay. To confirm the presence of PSP toxins and to obtain their profile, these samples were analyzed using an optimized version of the Official Method AOAC 2005.06 and using LC–MS/MS (HILIC). The presence of some PSP toxins (PSTs) in that geographical area (~600 km of coast) was confirmed for the first time. The estimated toxicities ranged from 170 to 983 µg STXdiHCl eq.·kg^−1^ for the AOAC 2005.06 method and from 150 to 1094 µg STXdiHCl eq.·kg^−1^ for the LC–MS/MS method, with a good correlation between both methods (r^2^ = 0.94). Most samples contained STX, GTX2,3, and GTX1,4, and some also had NEO and dcGTX2. All of the PSP-positive samples also contained gymnodimine A, with the concentrations of the two groups of toxins being significantly correlated. The PSP toxin profiles suggest that a species of the genus *Alexandrium* was likely the causative agent. The presence of gymnodimine A suggests that *A. ostenfeldii* could be involved, but the contribution of a mixture of *Alexandrium* species cannot be ruled out.

## 1. Introduction

Paralytic shellfish toxins (PSTs) are a group of natural neurotoxic alkaloids that, in the marine environment, are produced by some bloom-forming dinoflagellate species, particularly several species of the genus *Alexandrium*, *Gymnodinium catenatum*, and *Pyrodinium bahamense*, with most of them distributed worldwide [1]. Up until now, more than 50 analogues of this group of toxins have been described with different toxicities, with saxitoxin (STX) being the most representative compound due to both its potent toxicity and early characterization [2]. The basic chemical structure of these toxins is based on a 3,4,6-trialkyl tetrahydropurine skeleton, and according to their substituents side-chains, they are classified into five main groups: carbamate, *N*-sulfocarbamoyl, decarbamoyl, 13-deoxycarbamoyl, and hydroxybenzoate toxins [3,4]. Not all of those toxins are equally toxic, and the toxicities relative to that of STX (toxicity equivalency factors, TEF) for the most important ones have been estimated by Oshima [5] and re-evaluated by EFSA [6].

When paralytic shellfish toxins (PSTs)-producing phytoplankton species develop, the toxins are mainly accumulated by filter-feeding organisms, in most cases without noticeable adverse effects on them [7,8,9]. Bivalve mollusks are the predominant accumulative species, although PSTs have also been detected in some gastropods, crustaceans, and less frequently in fish [1,10]. Upon ingestion of contaminated shellfish, these toxins are responsible for the paralytic shellfish poisoning (PSP) syndrome in humans, characterized by symptoms such as tingling sensation around the lips, gums, and tongue; numbness of the extremities; and in extreme cases, fatalities due to respiratory muscle paralysis. The allowable limit of PSP toxicity in live bivalve mollusks has been established within the EU as 800 µg STXdiHCl eq kg^−1^ (measured in the whole body or any part edible separately) [11,12].

PSTs have been traditionally monitored using a mouse bioassay (MBA). This method has ethical and technical problems, which led the EU to replace it with the “Official method AOAC 2005.06” as the reference method [13] for the analysis of this group of toxins. The method, developed by Lawrence et al. [14,15], is based on pre-column derivatization, followed by HPLC separation and fluorescence detection of derivatives (some of which are shared by several toxins). This method has some drawbacks, with the most important being the production of the same derivatives by different toxins, which makes it impossible to quantify some compounds separately (especially some isomers), and the long time required to complete an analysis.

Recently, a method that uses LC tandem mass spectrometry (MS/MS) has been implemented as an alternative to LC–FLD for the determination of PSTs in shellfish. This methodology allows for the full separation and quantification of epimeric pairs and reduces the uncertainty of the quantification, which are the main weaknesses of the current EU reference method [16].

PSP toxicity has been detected in many places along the Atlantic coast of Europe [17], with two main causative organisms, *Gymnodinium catenatum* and *Alexandrium* spp. The first species sporadically affects the western coast of Spain and Portugal [18,19,20,21,22]. The organisms of the genus *Alexandrium*, nevertheless, affect many countries, including France, Ireland, the United Kingdom, Denmark, Iceland, Portugal, and Norway [23,24,25,26,27,28,29,30]. Notwithstanding, in two areas, comprising Belgium, The Netherlands, and Germany in the North, and most of Cantabrian coast and the southern part of the French Bay of Biscay (~600 km) in the South, PSP-producing *Alexandrium* had not previously been detected. In 2018 and 2019, PSP toxicity was detected in a few samples using the monitoring system of the Basque Country (and, in 2019, in one sample from Cantabria) and using the MBA [31]. Some organisms of the genus *Alexandrium* have been shown to produce lipophilic toxins [32,33,34,35,36,37] but, in most cases, not concurrently with PSP ones. *Alexandrium ostenfeldii* has been shown to produce PSTs together with cyclic imines (spirolides and/or gymnodimines) [33,38,39,40].

**Figure 1:**
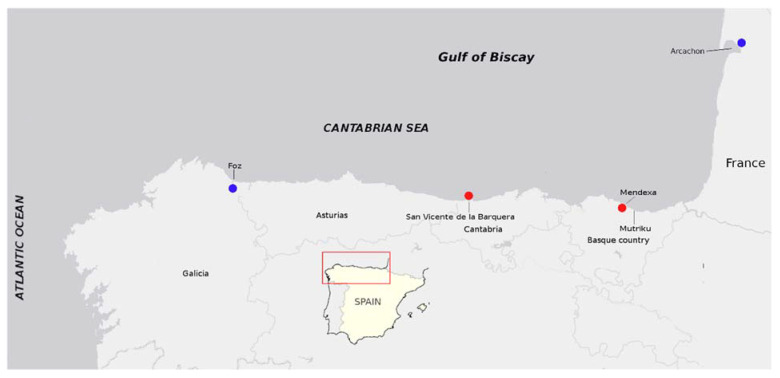
Locations from which PSTs had been reported in this study (red dots) and the closest ones previously cited (blue dots) from the Gulf of Biscay.

This study confirms the presence (in 2018 and 2019) of PSTs in bivalves from the Spanish Cantabrian Coast (the Basque Country and Cantabria) (Figure 1) and focuses on the determination of their toxin profile, their estimated toxicity (from an optimized variation of the official method AOAC 2005.06 (liquid chromatography-fluorescence detection, LC–FLD; and by a liquid chromatography-triple quadrupole mass spectrometry, LC–MS/MS, method), and the possible co-occurrence with cyclic imines.

## 2. Results

Mussel samples were routinely collected by the monitoring systems of Cantabria and the Basque Country, in several geographic locations, with weekly or fortnightly frequency, and were immediately tested via the MBA. Since 2002, the PSP toxicity was determined in 2118 mussel samples from the area (distribution by month is shown in the Supplementary Materials Figure S1), resulting in only 12 positive results (>380 µg STXdiHCl eq.·kg^−1^) and 3 samples above the regulatory limit (800 µg STXdiHCl eq.·kg^−1^). All samples but one came from Mendexa and Mutriku, in the Basque Country. The other was from San Vicente de la Barquera, in Cantabria. The presence of PSTs was detected in the subset of samples kept for confirmation (six samples collected in 2018 and 2019).

**Figure 2:**
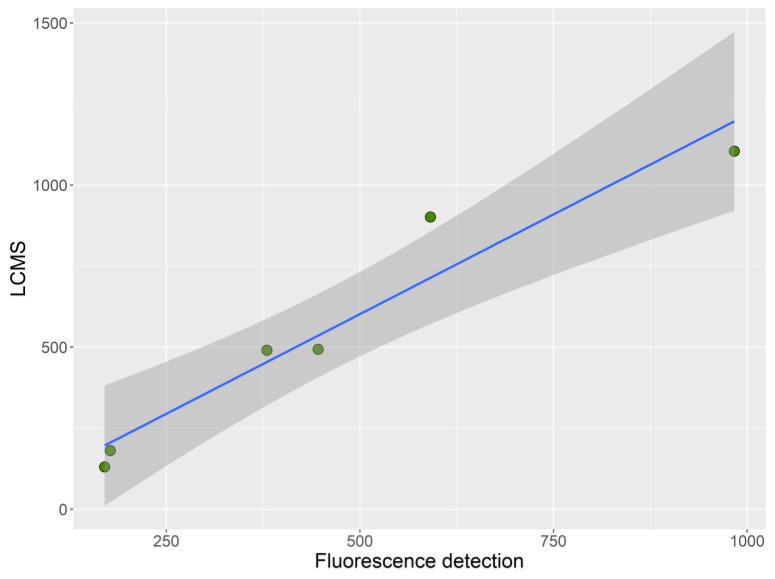
Toxicity (µg STXdiHCl eq.·kg^−1^) of the collected samples estimated by liquid chromatography-fluorescence detection (LC–FLD) and by liquid chromatography-triple quadrupole mass spectrometry (LC–MS/MS).

Only two of the three samples that surpassed the regulatory limit set by MBA, both from Mendexa, were analyzed. The toxicity estimates made by the two analytical methods (LC–FLD and LC–MS/MS), coincided well with the MBA toxicities (Figure 2 and Table S1).

**Figure 3:**
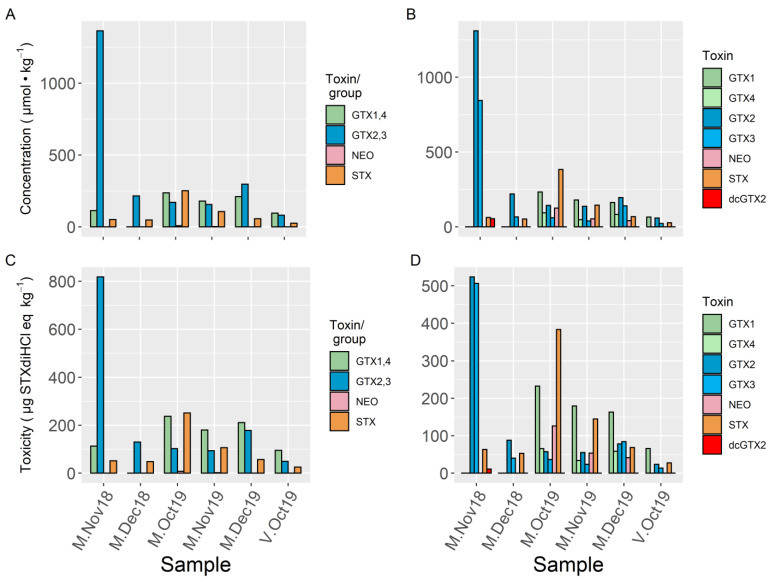
Toxin concentrations (**A**,**B**) and PSP toxicities (**C**,**D**) estimated for each toxin or group of epimers from the analyses of naturally contaminated mussels from Mendexa (M) and San Vicente de la Barquera (V) using LC–FLD (**A**,**C**) and LC–MS/MS (**B**,**D**). The LOQ for GTX1,4 found using LC–FLD is high (144 µg STXdiHCl eq.·kg^−1^), so we report the values higher than LOD for this pair of toxins even when they were below the LOQ.

**Figure 4:**
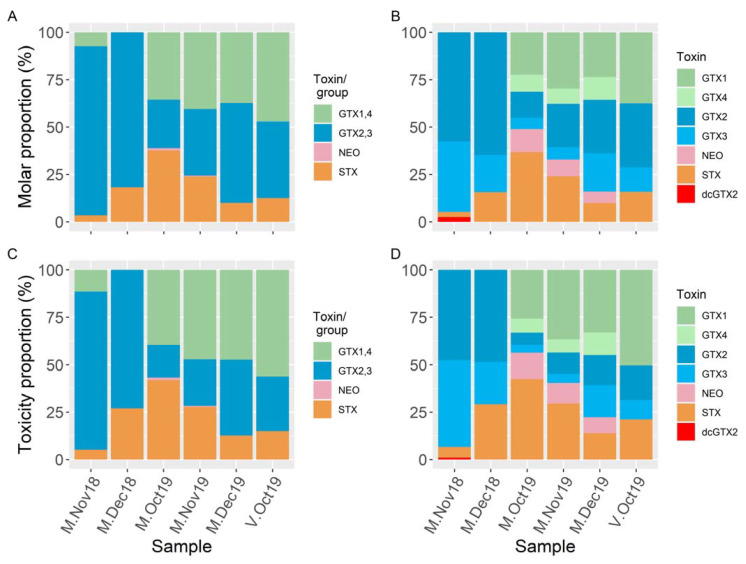
Comparison of PSP toxin profiles, in molar basis, (**A**,**B**) and toxicity, in STXdiHCl eq, (**C**,**D**), of the mussel samples collected from the Cantabrian and Basque coasts, quantified by LC–FLD (**A**,**C**) and LC–MS/MS (**B**,**D**). For LC–FLD, groups of toxins are shown instead of individual toxins when the toxins in the group cannot be individually analyzed. Similar colors were used for toxins that were quantified jointly by LC–FLD.

All of the samples collected from Mendexa and San Vicente de la Barquera showed similar toxin profiles, which only included STX and the groups GTX1,4, and GTX2,3 when analyzed by LC–FLD (Figure 3 and Figure 4). Using LC–MS/MS, in addition to the toxins belonging to the groups detected by LC–FLD, dcGTX2 has been found in three samples (two below LOQ). NEO has been detected below LOQ by LC–FLD, and it was confirmed and quantified by LC–MS/MS in three samples. It was also observed that the two toxins of each group of epimers detected by LC–FLD were present in the samples, with the only exception being one sample (V-Oct19) in which GTX4 was not detected.

In terms of their toxicity, estimated by LC–FLD, the dominant toxins in the sample from San Vicente de la Barquera were GTX1,4 (56%) followed by GTX2,3 (29%) and STX (15%) (Figure 4) (the estimate of GTX1,4 could have a large uncertainty because it was lower than the nominal LOQ for the LC–FLD method) (Table S2). In the samples from Mendexa, the proportions of the toxicities due to the detected toxins were different in 2018 and 2019. In 2018, GTX2,3 constituted 73–83% of the toxicity, STX constituted 5–27%, and GTX1,4 constituted less than 12%. In 2019, GTX1,4 and STX were more important, representing 36–56% and 12–38% of the total toxicity, respectively; and GTX2,3 was less important, representing 15–37% of the total toxin; and very small contributions of NEO (<LOQ) were detected. By LC–MS/MS, the toxicity profiles were similar, but some differences can be observed. dcGTX2 was detected, and the contribution of NEO to the total toxicity was noticeably larger (due to a greater sensitivity). LC–MS/MS showed that the α-epimers (GTX1 and 2) were more abundant than the β epimers (GTX4 and 3) (with the exception of M-Nov18). The estimated toxicities of the toxins or groups of epimeric toxins estimated by LC–FLD coincided well with the estimations made by LC–MS/MS, the slopes were near 1, and good correlation coefficients were obtained by model II linear regression: 0.96 for GTX1,4 (when the values below LOQ are excluded); 0.99 for GTX2,3; and 0.99 for STX.

The proportions between toxin concentrations (Figure 4A,B) are similar to those between their estimated toxicities (Figure 4C,D), with the most important differences being the increase in the contributions of GTX2 and GTX3, and the decrease in the contributions of GTX1, GTX4, and STX.

**Figure 5:**
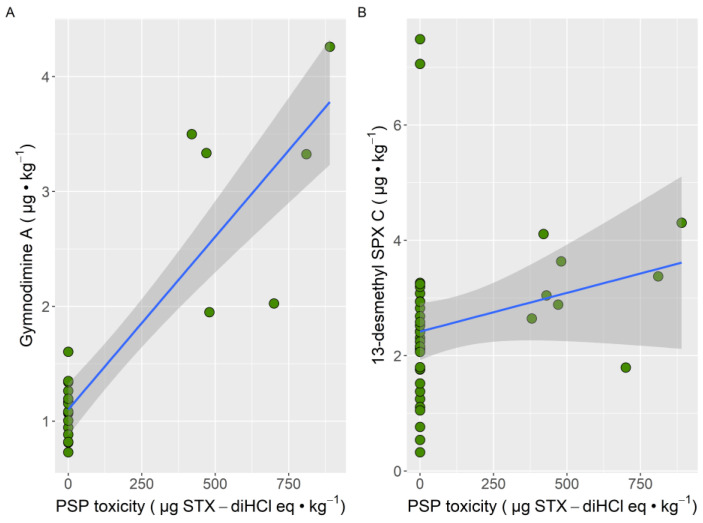
Relationship between the PSP toxicity with gymnodimine A (**A**) and 13-desmethyl spirolide C (**B**) concentrations in the mussel samples obtained from the Cantabrian Coast. The blue line is the regression line, and the shadowed area represents the limits of confidence for the regression.

All samples that contained PSTs also contained gymnodimine A, and all but one also contained 13-desmethyl spirolide C. The relationship of PSP with gymnodimine A seems to be stronger than with 13-desmethyl spirolide C (Figure 5).

## 3. Discussion

This is the first report on PSP toxins from an area comprising ~600 km of the Cantabrian coasts, from the Ría of Foz, in Galicia (NW Spain) to Arcachon (SW France) [17,41].

The toxicity levels observed and the very low prevalence of this type of toxicity suggest a low risk for mollusk fisheries or aquaculture in the area. However, in a few samples, the PSP toxicity exceeded the regulatory limit, consequently becoming a risk for human consumers and demonstrating the importance of an efficient monitoring system.

The relationship observed between concentrations of gymnodimine A and PSTs in the mussel samples points to *A. ostenfeldii* as the species responsible for the two toxins. This species was found in Mendexa during the first detection of PSP toxicity [31] but in such a low concentration (80 cells L^−1^) that it cannot explain the relatively high PST levels detected in the mussels from the area. In the Northern Baltic Sea, a similar toxin composition has been found in water samples collected and in *A. ostenfeldii* cultures isolated from the Åland archipelago, between Finland and Sweden [38,42,43,44]. *A. ostenfeldii* can also produce spirolides (reviewed in [38]), but the relatively high levels of 13-desmethyl spirolide C, frequently found in the studied area of the Cantabrian Sea without the concurrent presence of PSTs, suggests that the production of the three groups of toxins by the same species is unlikely. Furthermore, it was suggested in a study of cyclic imines that included, among others, the samples used in this work that several species could be involved in the production of gymnodimine A and 13-desmethyl spirolide C given the existence of two groups of samples with different gymnodimine A/13-desmethyl spirolide C ratios [45]. Nevertheless, the possibility that PSTs and gymnodimine were produced (each of them) by different dinoflagellate species and accumulated by mussels cannot be ruled out. In such a case, *A. minutum* could be responsible for the PST production. The PSP toxin profile of the samples in this study is similar to that reported for the *A. minutum* strains from Galicia (Northwest coast of Spain), the closest location in which PSTs were detected in Spain [46]. The Galician profile is characterized by the presence of GTX1,4 as the major toxin and by GTX1,2, and STX, although in Galician studied strains of *A. minutum*, STX was not detected. This non-STX producing strain has also been observed in the *A. minutum* isolated from the central coast of Portugal [47] and in different locations of the Mediterranean sea, such as Mallorca, Sardinia, and Sicily [48]. In the coastal waters of Greece, only GTX1,4 has been associated with *A. minutum* blooms [49] while clones from the Gulf of Trieste (Italy) showed the same profile reported in this study (GTX1,4, GTX2,3, and STX) [50]. These differences might be explained, for example, by the partial conversion of the GTX into STX by biotransformation mechanisms produced by toxin-transforming enzymes or certain marine bacteria [51,52,53]. The predominance of GTX2,3 and STX in the samples analyzed in 2018 is unusual in the Iberian Peninsula; however, this toxin profile has been described in mussel samples from Arcachon [54], the closest location in which PSTs were detected in France (the causative species has not been identified [41]), and in *A. minutum* strains isolated from the southern coast of England [28]. The other species known to produce PSTs in the Iberian Peninsula, *Gymnodinium catenatum*, has a much more complex toxin profile [20,55], which includes sulfocarbamoyl toxins and has never been detected in the Cantabrian Sea or in any other point of the Atlantic Ocean north of Cape Finisterre.

The season in which the studied PSP episodes appeared, late autumn, is atypical for *A. minutum* episodes on most of the Atlantic coast of Europe, with blooms usually in spring or summer [56], as well as in Galicia (typically in June and late August–early September). In Arcachon, however, the risk of PSP is considered high not only in summer but also in December [57], suggesting that the causative organisms could be the same or closely related.

*Alexandrium* cells have been detected in the area in spring and summer but also, rarely, in September and January [58,59]. Unfortunately, they have not been identified at the species level.

## 4. Materials and Methods

### 4.1. Standards, Solvents, and Reagents

The certified reference materials saxitoxin (STX), neosaxitoxin (NEO), decarbamoylsaxitoxin (dcSTX), decarbamoylneosaxitoxin (dcNEO), gonyautoxin 5 (GTX5), gonyautoxin 6 (GTX6), gonyautoxins 1 and 4 (GTX1,4), gonyautoxins 2 and 3 (GTX2,3), decarbamoylgonyautoxins 2 and 3 (dcGTX2,3), *N*-sulfocarbamoyl-gonyautoxins 2 and 3 (C1,2), and gymnodimine A were obtained from the Institute of Biotoxin Metrology, National Research Council Canada (NRCC, Halifax, NS, Canada). All of these toxins, gonyautoxin 6 (GTX6), and 13-desmethyl spirolide C were also acquired from Cifga S.A. (Lugo, Spain).

LC–MS grade methanol was purchased from Honeywell (Charlotte, NC, USA). Acetonitrile; LC–MS grade, glacial acetic acid; ammonium acetate (96%); sodium chloride (99.5%); di-sodium hydrogen phosphate (99%); and hydrogen peroxide solution (30%) were obtained from Merck (Darmstadt, Germany). Periodic acid (99.5%) and ammonium formate (98.1%) were from VWR (Radnor, PA, USA), and sodium hydroxide (98%) was from Panreac (Castellar del Vallès, Barcelona, Spain). Ammonium hydroxide (NH_4_OH, 25%) and formic acid were obtained from Merck (Barcelona, Spain). Ultrapure water was obtained from a Milli-Q Gradient A-10 system (Millipore, Burlington, MA, USA).

The reversed-phase Supelclean LC-18 (500 mg/3 mL) and graphitized carbon Supelclean ENVICarb (250 mg/3 mL) cartridges were acquired from Supelco (Bellefonte, PA, USA). Mixed-mode Strata-X-CW (60 mg/3 mL) were supplied by Phenomenex (Torrance, CA, USA). Hydrophilic PES 0.22 µm Millex-GP syringe filters were purchased from Millipore (Burlington, MA, USA), and Nylon membrane filters, 0.22 µm, were from Filter-Lab (Sant Pere de Riudebitlles, Barcelona, Spain).

### 4.2. Shellfish Sampling and Tissue Preparation

Previous to this work and in the framework of the monitoring systems of Cantabria and the Basque Country, the mussel samples (*Mytilus galloprovincialis*) were collected by AZTI in Mendexa, Mutriku (Basque Country), one or two times per month and by the Servicio de Actividades Pesqueras de la Dirección General de Pesca y Alimentación of the Cantabria Government in San Vicente de la Barquera (Cantabria) with a weekly frequency between March and September, and fortnightly during autumn and winter months. Approximately 100 g of mussel meat was rinsed with tap and ultrapure water to reduce the salt concentration and then homogenized using a blender.

Aliquots of the obtained homogenates were extracted for PSP MBA and the analysis of lipophilic toxins by LC–MS/MS. Six of the MBA-positive samples were distributed into plastic containers and kept at −20 °C until their analysis.

### 4.3. Sample Extraction and Preparation

#### 4.3.1. LC–FLD

The AOAC 2005.06 [34] procedure for the extraction of PSTs was applied as follows: (1) double-extraction with 1% acetic acid (first extraction at 100 °C), shaking for a few seconds and centrifugation; (2) extract clean-up by SPE C18; and (3) pH adjustment to 6.5 using an automated pH-meter (Metrohm Hispania, Madrid, Spain). A filtered step using syringe filters was added to remove possible interfering particles in the oxidation reaction. The matrix modifier for periodate oxidation was prepared following the same extraction and clean-up protocol.

C18 extracts were then subjected to oxidation with peroxide, and the oxidation products of the *N*-hydroxylated toxins (dcGTX2,3; C1,2; dcSTX; GTX2,3; GTX5; and STX) were subsequently analyzed. Non-*N*-hydroxylated toxins (GTX1,4; NEO; dcNEO; GTX6) were quantified in the fractions obtained by mixed-mode SPE cartridge fractionation [35], and oxidized using a periodate solution in the presence of matrix modifier.

#### 4.3.2. LC–MS/MS

The procedure proposed by Turner et al. [16] was used. As for LC–FLD, a double-extraction with 1% acetic acid (first extraction at 100 °C), shaking for few seconds and centrifugation was carried out. An aliquot (1 mL) of the supernatant was mixed with 5 µL of ammonium hydroxide (25% NH_4_OH), and 400 µL of the mixture was loaded onto a graphitized carbon cartridge Supelclean ENVICarb (250 mg/3 mL) conditioned with 3 mL of 20% MeCN in 1% AcOH, followed by 3 mL of 0.025% NH_4_OH. The cartridges were washed with 700 µL of Milli-Q water and eluted with 2 mL of 20%MeCN in 1% AcOH. Finally, 100 µL of the obtained eluate was diluted with 300 µL of MeCN placed into a propylene vial and analyzed by LC–MS/MS.

#### 4.3.3. Cyclic Imines

The cyclic imines were extracted from 2 g aliquots of the homogenate following the procedure of Lamas et al. [45] and the guidelines of the EU-RL [60]. In brief, the aliquot was vortexed with 9 mL of MeOH for 1 min and centrifuged at 2000× *g* (4 °C) for 10 min, and the pellet was re-extracted. Finally, the supernatants of the two extractions were combined, and the volume was made up to 20 mL with MeOH. An aliquot of the final extract was filtered through a PVDF 0.22 µm syringe filter (Agilent Technologies, Santa Clara, CA, USA) and diluted with MeOH (1:1 *v:v*) prior to injection into the LC/MS system.

### 4.4. LC–FLD Conditions

The PSP toxins were determined using an Agilent Technologies (Wilmington, DE, USA) UHPLC–FLD system. The instrument consisted of an Agilent 1290 Series LC system with a high-pressure binary pump, a multi-sampler set at 4 °C, and an oven for the LC column set at 35 °C. The fluorescence detector was an Agilent 1260 Infinity II using excitation and emission wavelengths of 340 nm and 395 nm, respectively. For the chromatographic separation, an Atlantis T3 column (75 mm × 2.1 mm, 3 µm) was used, connected to an Atlantis T3 Vanguard cartridge (5 mm × 2.1 mm, 3 µm) (Waters, Milford, MA, USA). The mobile phase consisted of 0.1 M ammonium formate (A) and 0.1 M ammonium formate with 10% acetonitrile (B), both adjusted to pH 6 ± 0.1 with 0.1 M acetic acid, filtered by a Nylon filter membrane, and sonicated for 15 min. A gradient program with a flow of 0.7 mL min^−1^ was run, starting with a 0.6 min isocratic at 5% B, followed by a linear increment to 45% B at 1.8 min. After an isocratic hold time of 2.2 min, in order to equilibrate the column, isocratic conditions at 0% B were held for 1.1 min, making the total run time 3.3 min. The injection volume was 15 µL. The version 2.4 of the OpenLab software (Agilent Technologies Spain, Madrid, Spain) was used to control the LC–FLD system and to acquire and process the data.

The LOQs of the method are given in Table S2 (Supplementary Materials).

### 4.5. LC–MS Conditions

#### 4.5.1. PSP Toxins

A sample analysis was performed on an Exion LC system (AB Sciex Spain, Madrid, Spain), which consisted of a quaternary pump, a vacuum degasser, an autosampler (AD multiplate sampler), and a temperature-controlled compartment for column (AD column oven) coupled with a triple quadrupole mass spectrometer, SCIEX TripleQuad™ 6500+ (AB Sciex Spain, Madrid, Spain), and equipped with an electrospray ion source (ESI). The analytes were separated using an Acquity UPLC Glycan BEH Amide (2.1 mm × 150 mm, 1.7 µm) column maintained at 60 °C. The autosampler temperature was set at 6 °C. The mobile phases were those proposed by Boundy et al. [61], which are A: water/formic acid/NH_4_OH (500/0.075/0.3 *v*/*v*/*v*); B: acetonitrile/water/formic acid (700/300/0.1 *v*/*v*/*v*). The gradient started at 0.4 mL min^−1^ with 4:96 A:B and was maintained for 3.5 min, followed by a linear change to reach 50:50 A:B at 6 min and by maintaining this composition until 7.5 min but increasing the flow rate to 0.5 mL min^−1^. The composition was then returned linearly to the initial conditions (96% B) in 0.5 min at 0.5 mL min^−1^; maintained for 1.5 min at the same conditions, the flow rate was increased again to 0.8 mL min^−1^ at 9.8 min and maintained for 0.6 min. Finally, the flow was returned to 0.4 mL min^−1^ for 0.5 min and maintained for 0.1 min. The total run time was 11 min, and the injection volume was 1 µL.

Quantitation was performed using multiple reaction monitoring (MRM) with a dwell time of 20 ms per transition. The MRM parameters for the PSP toxins are listed in Table S3. The optimized source parameters were as follows: 30 psi for curtain gas, medium for collision activation dissociation (CAD), 650 °C for turbo heater temperature, −4500 V (ESI−) or 5000 V (ESI+) for ion spray voltage, 80 psi for Gas 1, and 80 psi for Gas 2. Quadrupole 1 and quadrupole 3 were maintained at unit resolution. Data processing was performed using SCIEX OS software.

The transitions used for each toxin are given in Table S3 (Supplementary Materials).

#### 4.5.2. Cyclic Imines

The analyses were carried out with an Acquity UPLC H-Class system (Waters, Barcelona, Spain) coupled with a Waters Xevo TQ-S triple quadrupole mass spectrometer (Barcelona, Spain) and equipped with an electrospray ionization source (ESI) interface, which was operated in positive mode.

For the chromatographic separation, an Acquity UPLC BEH C18 (2.1 mm × 100 mm, 1.7 µm) (Waters, Barcelona, Spain) column was selected. The chromatographic conditions were as follows: 45 °C of column temperature; 400 µL∙min^−1^ of flow rate; and a binary gradient of mobile phase composed by phase A (water) and B (MeCN 90%), both with 6.7 mM NH_4_OH (approx. pH = 11). The chromatographic separation gradient started at 25% of phase B (held 1.66 min), followed by a linear increment to 95% B at 4.3 min and maintaining this composition for 6.28 min. Finally, the gradient was returned linearly to the initial conditions in 2 min and maintained for 9 min to equilibrate the column for the next injection. The injection volume was 5 µL.

The determination was carried out in MRM mode [M + H]^+^ by selecting two transitions. For gymnodimine A, 508.3 > 490.2 (collision energy, 22 eV) and 508.3 > 162.2 (collision energy, 42 eV) were selected as the quantifier and qualifier, respectively, both with 50 V of cone voltage. For 13-desmethyl spirolide C, the selected transitions were as follows: 692.5 > 164.3 (collision energy, 42 eV) and 692.5 > 444.3 (collision energy, 36 eV) as the quantifier and qualifier transitions, respectively, both with 60 V of cone voltage.

The electrospray interface parameters were as follows: capillary voltage = 1 V, solvation temperature = 450 °C, N2 flow = 850 L∙h^−1^, and cone gas flow = 150 L∙h^−1^.

Gymnodimine A was identified by comparison with a reference certified solution of this toxin. The quantification was carried out using different working standard solutions obtained by dilution in MeOH–water (1:1 *v:v*) of the reference certified solution.

The linearity of the method is good (R^2^ > 0.99), as was the gymnodimine A recoveries, which were between 93 and 97%, depending on the species. The limits of quantification (LOQ) and detection (LOD) were 0.25 and 0.075 µg∙kg^−1^, respectively.

### 4.6. Toxicity Computation

Three estimates of the total PSP toxicity were obtained. The first was provided by the monitoring system and directly determined by the MBA. The second was computed from the concentrations estimated by LC–MS/MS. The concentration of each toxin was multiplied by its Toxicity Equivalent Factor (TEF) [6], and finally, the toxicities of all toxins were added to give the total toxicity. The third estimate was made using the toxin concentrations estimated by LC–FLD. The procedure was the same as for LC–MS/MS but, for the toxins that cannot be individually analyzed (GTX 1, and GTX 2 and 3), the TEF corresponding to the most abundant component of each pair was used.

### 4.7. Statistical Analysis

The regression analyses were carried out using R [62] and the R package smart 3 [63]. Plots were generated with the R package GGPLOT2 [64].

## Supplementary Materials

The following are available online at https://www.mdpi.com/article/10.3390/toxins13110761/s1, Table S1: Toxicities estimated by MBA, LC–FLD, and LC–MS/MS, Table S2: Limits of quantification (LOQs) calculated for each PST, Table S3: MS/MS fragmentation conditions for paralytic shellfish toxin determination, Figure S1. Distribution per month of samples analyzed by MBA using the monitoring system.

## References

[1] Deeds J.R., Landsberg J.H., Etheridge S.M., Pitcher G.C., Longan S.W. (2008). Non-traditional vectors for paralytic shellfish poi-soning. Mar. Drugs.

[2] Schantz E.J. (1986). Chemistry and Biology of Saxitoxin and Related Toxins. Ann. N. Y. Acad. Sci..

[3] Llewellyn L.E. (2006). Saxitoxin, a toxic marine natural product that targets a multitude of receptors. Nat. Prod. Rep..

[4] Wiese M., D’Agostino P.M., Mihali T.K., Moffitt M.C., Neilan B.A. (2010). Neurotoxic Alkaloids: Saxitoxin and Its Analogs. Mar. Drugs.

[5] Oshima Y., Hallegraeff G.M., Anderson D.M., Cembella A.D. (1995). Post-column Derivatization HPLC Methods for Paralytic Shellfish Poisons. Manual on Harmful Marine Microalgae.

[6] EFSA Panel on Contaminants in the Food Chain (2009). Scientific Opinion of the Panel on Contaminants in the Food Chain on a request from the European Commission on Marine Biotoxins in Shellfish—Saxitoxin Group. EFSA J..

[7] Bricelj V.M., Connell L., Konoki K., MacQuarrie S.P., Scheuer T., Catterall W.A., Trainer V.L. (2005). Sodium channel mutation leading to saxitoxin resistance in clams increases risk of PSP. Nat. Cell Biol..

[8] Bricelj V.M., Shumway S.E. (1998). Paralytic Shellfish Toxins in Bivalve Molluscs: Occurrence, Transfer Kinetics, and Biotransformation. Rev. Fish. Sci..

[9] Haberkorn H., Lambert C., Le Goïc N., Quéré C., Bruneau A., Riso R., Auffret M., Soudant P. (2014). Cellular and biochemical responses of the oyster *Crassostrea gigas* to controlled exposures to metals and *Alexandrium minutum*. Aquat. Toxicol..

[10] Ben-Gigirey B., Rossignoli A.E., Riobó P., Rodríguez F. (2020). First Report of Paralytic Shellfish Toxins in Marine Invertebrates and Fish in Spain. Toxins.

[11] EC (2004). Regulation (EC) No 853/2004 of the European Parliament and of the Council of 29 April 2004 laying down specific hygiene rules for food of animal origin. Off. J. Eur. Union.

[12] EC (2021). Commission Delegated Regulation (EU) 2021/1374 of 12 April 2021 Amending Annex III to Regulation (EC) No 853/2004 of the European Parliament and of the Council on Specific Hygiene Requirements for Food of Animal Origin (Text with EEA Rele-Vance). Off. J. Eur. Union.

[13] EC (2019). COMMISSION IMPLEMENTING REGULATION (EU) 2019/627 of 15 March 2019 laying down uniform practical arrangements for the performance of official controls on products of animal origin intended for human consumption in accordance with Regulation (EU) 2017/625 of the European Parliament and of the Council and amending Commission Regulation (EC) No 2074/2005 as regards official controls. Off. J. Eur. Union.

[14] Lawrence J.F., Menard C., Charbonneauc C.F. (1991). A study of ten toxins associated whit paralytic shellfish poison using pre-chromatographic oxidation and liquid chromatography whit fluorescence detection. J. Assoc. Off. Anal. Chem..

[15] Lawrence J.F., Niedzwiadek B., Menard C. (2005). Quantitative Determination of Paralytic Shellfish Poisoning Toxins in Shellfish Using Prechromatographic Oxidation and Liquid Chromatography with Fluorescence Detection: Collaborative Study. J. AOAC Int..

[16] Turner A.D., Dhanji-Rapkova M., Fong S.Y.T., Hungerford J., McNabb P.S., Boundy M.J., Harwood D.T., Aanrud S., Alfonso C., Alvarez M. (2020). Ultrahigh-Performance Hydrophilic Interaction Liquid Chromatography with Tandem Mass Spectrometry Method for the Determination of Paralytic Shellfish Toxins and Tetrodotoxin in Mussels, Oysters, Clams, Cockles, and Scallops: Collaborative Study. J. AOAC Int..

[17] Bresnan E., Arévalo F., Belin C., Branco M.A.C., Cembella A.D., Clarke D., Correa J., Davidson K., Dhanji-Rapkova M., Lozano R.F. (2021). Diversity and regional distribution of harmful algal events along the Atlantic margin of Europe. Harmful Algae.

[18] Anderson D.M., Sullivan J.J., Reguera B. (1989). Paralytic shellfish poisoning in northwest Spain: The toxicity of the dinoflagellate Gymnodinium catenatum. Toxicon.

[19] Mamán L., Fernández R., Jaén D., Mata A.J., Morales J., Jiménez C., Márquez I., Norte M., Fernández J.J. (2004). Estudio de las proliferaciones del dino-flagelado *Gymnodinium catenatum* (Graham) en la costa de Andalucía (Sur Península Ibérica). VIII Reunión Ibérica sobre Fi-toplacton Tóxico y Biotoxinas.

[20] Ordas M.C., Fraga S., Franco J.M., Ordas A., Figueras A. (2004). Toxin and molecular analysis of *Gymnodinium catenatum* (Di-nophyceae) strains from Galicia (NW Spain) and Andalucia (S Spain). J. Plankton Res..

[21] Rodrigues S.M., de Carvalho M., Mestre T., Ferreira J.J., Coelho M., Peralta R., Vale P. (2012). Paralytic shellfish poisoning due to ingestion of *Gymnodinium catenatum* contaminated cockles—Application of the AOAC HPLC Official Method. Toxicon.

[22] Bravo I., Fraga S., Figueroa R.I., Pazos Y., Massanet A., Ramilo I. (2010). Bloom dynamics and life cycle strategies of two toxic dinoflagellates in a coastal upwelling system (NW Iberian Peninsula). Deep. Sea Res. Part II Top. Stud. Oceanogr..

[23] Touzet N., Franco J.M., Raine R. (2007). Characterization of nontoxic and toxin-producing strains of *Alexandrium minutum* (Di-nophyceae) in Irish coastal waters. Appl. Environ. Microbiol..

[24] Touzet N., Farrell H., Rathaille A.N., Rodriguez P., Alfonso A., Botana L.M., Raine R. (2010). Dynamics of co-occurring *Alexandrium minutum* (Global Clade) and A. tamarense (West European) (Dinophyceae) during a summer bloom in Cork Harbour, Ireland (2006). Deep. Sea Res. Part II Top. Stud. Oceanogr..

[25] Vale P., Botelho M.J., Rodrigues S.M., Gomes S.S., Sampayo M.A.d.M. (2008). Two decades of marine biotoxin monitoring in bi-valves from Portugal (1986–2006): A review of exposure assessment. Harmful Algae.

[26] Nascimento S.M., Purdie D.A., Lilly E.L., Larsen J., Morris S. (2005). Toxin Profile, Pigment Composition, and Large Subunit rDNA Phylogenetic Analysis of an *Alexandrium minutum* (Dinophyceae) Strain Isolated from the Fleet Lagoon, United Kingdom. J. Phycol..

[27] Hansen G., Daugbjerg N., Franco J.M. (2003). Morphology, toxin composition and LSU rDNA phylogeny of *Alexandrium minutum* (Dinophyceae) from Denmark, with some morphological observations on other European strains. Harmful Algae.

[28] Burrell S., Gunnarsson T., Gunnarsson K., Clarke D., Turner A.D. (2013). First detection of paralytic shellfish poisoning (PSP) toxins in Icelandic mussels (*Mytilus edulis*): Links to causative phytoplankton species. Food Control..

[29] Karlson B., Andersen P., Arneborg L., Cembella A., Eikrem W., John U., West J.J., Klemm K., Kobos J., Lehtinen S. (2021). Harmful algal blooms and their effects in coastal seas of Northern Europe. Harmful Algae.

[30] Lassus P., Baron R., Garen P., Truquet P., Masselin P., Bardouil M., Leguay D., Amzil Z. (2004). Paralytic shellfish poison outbreaks in the Penzé estuary: Environmental factors affecting toxin uptake in the oyster, *Crassostrea gigas*. Aquat. Living Resour..

[31] Ferrer L., Revilla M., Laza-Martínez A., Sagarminaga Y., Fontán A., Larreta J., Zorita I., Solaun O., Rodríguez J.G., Arantzamendi L. (2019). Occurrence of the toxic dinoflagellate *Alexandrium ostenfeldii* in the coastal waters of the southeastern Bay of Biscay. Front. Mar. Sci..

[32] Cembella A.D., Lewis N.I., Quilliam M.A. (1999). Spirolide composition of micro-extracted pooled cells isolated from natural plankton assemblages and from cultures of the dinoflagellate *Alexandrium ostenfeldii*. Nat. Toxins.

[33] Van Wagoner R.M., Misner I., Tomas C.R., Wright J.L.C. (2011). Occurrence of 12-methylgymnodimine in a spirolide-producing dinoflagellate *Alexandrium peruvianum* and the biogenetic implications. Tetrahedron Lett..

[34] Tillmann U., Krock B., Wietkamp S., Beran A. (2020). A Mediterranean *Alexandrium taylorii* (Dinophyceae) Strain Produces Goniodomin A and Lytic Compounds but Not Paralytic Shellfish Toxins. Toxins.

[35] Harris C.M., Reece K.S., Harris T.M. (2020). Revisiting the toxin profile of *Alexandrium pseudogonyaulax*; Formation of a desmethyl congener of goniodomin A. Toxicon.

[36] Hsia M.H., Morton S.L., Smith L.L., Beauchesne K.R., Huncik K.M., Moeller P.D.R. (2006). Production of goniodomin A by the planktonic, chain-forming dinoflagellate *Alexandrium monilatum* (Howell) Balech isolated from the Gulf Coast of the United States. Harmful Algae.

[37] Krock B., Tillmann U., Wen Y.Y., Hansen P.J., Larsen T.O., Andersen A.J.C. (2018). Development of a LC-MS/MS method for the quantification of goniodomins A and B and its application to *Alexandrium pseudogonyaulax* strains and plankton field samples of Danish coastal waters. Toxicon.

[38] Salgado P., Riobo P., Rodríguez F., Franco J.M., Bravo I. (2015). Differences in the toxin profiles of *Alexandrium ostenfeldii* (*Di-nophyceae*) strains isolated from different geographic origins: Evidence of paralytic toxin, spirolide, and gymnodimine. Toxicon.

[39] Zurhelle C., Nieva J., Tillmann U., Harder T., Krock B., Tebben J. (2018). Identification of Novel Gymnodimines and Spirolides from the Marine Dinoflagellate *Alexandrium ostenfeldii*. Mar. Drugs.

[40] Cembella A.D., Lewis N.I., Quilliam M. (2000). The marine dinoflagellate *Alexandrium ostenfeldii* (*Dinophyceae*) as the causative organism of spirolide shellfish toxins. Phycologia.

[41] Belin C., Soudant D. (2018). Trente Années d’Observation des Micro-Algues et des Toxines d’Algues sur le Littoral.

[42] Riobó P., Rodriguez F., Garrido J.L., Franco J.M. Toxin profiles of *A. ostenfeldii* and *A. peruvianum*. Comparison of two clonal strains with different light tolerance. Marine and Freshwater Toxins Analysis. Proceedings of the Fourth Joint Symposium and AOAC Task Force Meeting.

[43] Hakanen P., Suikkanen S., Franzén J., Franzén H., Kankaanpää H., Kremp A. (2012). Bloom and toxin dynamics of *Alexandrium ostenfeldii* in a shallow embayment at the SW coast of Finland, northern Baltic Sea. Harmful Algae.

[44] Kremp A., Lindholm T., Dreßler N., Erler K., Gerdts G., Eirtovaara S., Leskinen E. (2009). Bloom forming *Alexandrium ostenfeldii* (*Dinophyceae*) in shallow waters of the Åland Archipelago, Northern Baltic Sea. Harmful Algae.

[45] Lamas J., Arévalo F., Moroño Á., Correa J., Rossignoli A.E., Blanco J. (2021). Gymnodimine A in mollusks from the north Atlantic Coast of Spain: Prevalence, concentration, and relationship with spirolides. Environ. Pollut..

[46] Franco J.M., Fernandez P., Reguera B. (1994). Toxin profiles of natural populations and cultures of *Alexandrium minutum* Halim from Galician (Spain) coastal waters. Environ. Boil. Fishes.

[47] Cembella A.D., Sullivan J.J., Boyer G.L., Taylor F.J.R., Andersen R.J. (1987). Variation in paralytic shellfish toxin composition within the *Protogonyaulax tamarensis/catenella* species complex; red tide dinoflagellates. Biochem. Syst. Ecol..

[48] Vila M., Giacobbe M.G., Masó M., Gangemi E., Penna A., Sampedro N., Azzaro F., Camp J., Galluzzi L. (2005). A comparative study on recurrent blooms of *Alexandrium minutum* in two Mediterranean coastal areas. Harmful Algae.

[49] Ignatiades L., Gotsisskretas O., Metaxatos A. (2007). Field and culture studies on the ecophysiology of the toxic dinoflagellate *Alex-andrium minutum* (Halim) present in Greek coastal waters. Harmful Algae.

[50] Yang I., Beszteri S., Tillmann U., Cembella A., John U. (2011). Growth- and nutrient-dependent gene expression in the toxigenic marine dinoflagellate *Alexandrium minutum*. Harmful Algae.

[51] Shimizu Y., Yoshioka M. (1981). Transformation of Paralytic Shellfish Toxins as Demonstrated in Scallop Homogenates. Science.

[52] Kotaki Y. (1989). Screening of Bacteria which Convert Gonyautoxin 2, 3 to Saxitoxin. Nippon. Suisan Gakkaishi.

[53] Kotaki Y., Oshima Y., Yasumoto T. (1985). Bacterial transformation of paralytic shellfish toxins in coral reef crabs and a marine snail. Nippon. Suisan Gakkaishi.

[54] Amzil Z., Quilliam M.A., Hu T., Wright J.L.C. (1999). Winter accumulation of paralytic shellfish toxins in digestive glands of mussels from Arcachon and Toulon (France) without detectable toxic plankton species revealed by interference in the mouse bioassay for lipophilic toxins. Nat. Toxins.

[55] Costa P.R., Robertson A., Quilliam M.A. (2015). Toxin Profile of *Gymnodinium catenatum* (*Dinophyceae*) from the Portuguese Coast, as Determined by Liquid Chromatography Tandem Mass Spectrometry. Mar. Drugs.

[56] Guallar C., Bacher C., Chapelle A. (2017). Global and local factors driving the phenology of *Alexandrium minutum* (*Halim*) blooms and its toxicity. Harmful Algae.

[57] Neaud-Masson N., Gautier E. (2021). Lieux et Périodes à Risque Pour les Toxines Paralysantes dans les Coquillages des Côtes Françaises; Note d’Information—Données REPHYTOX-Ifremer, Banque Quadrige. https://archimer.ifremer.fr/doc/00692/80445/83582.pdf.

[58] Muñiz O., Revilla M., Rodríguez M.I.R., Laza-Martínez A., Seoane S., Franco J., Orive E. (2017). Evaluation of phytoplankton quality and toxicity risk based on a long-term time series previous to the implementation of a bivalve farm (Basque coast as a case study). Reg. Stud. Mar. Sci..

[59] Bilbao J., Muñiz O., Revilla M., Rodríguez J.G., Laza-Martínez A., Seoane S. (2020). Suitability of two areas of the Basque coast to sustain shellfish aquaculture according to both the presence of potentially toxic phytoplankton and the biotoxins regulated by the European Union. Reg. Stud. Mar. Sci..

[60] EURLMB (2014). EU-Harmonised Standard Operating Procedure for Determination of Lipophilic Marine Biotoxins in Molluscs by LC-MS/MS.Version 5. https://www.aesan.gob.es/AECOSAN/docs/documentos/laboratorios/LNRBM/ARCHIVO2EU-Harmonised-SOP-LIPO-LCMSMS_Version5.pdf.

[61] Boundy M.J., Selwood A.I., Harwood D.T., McNabb P.S., Turner A.D. (2015). Development of a sensitive and selective liquid chromatography–mass spectrometry method for high throughput analysis of paralytic shellfish toxins using graphitised carbon solid phase extraction. J. Chromatogr. A.

[62] R Core Team (2019). R: A Language and Environment for Statistical Computing.

[63] Warton D.I., Duursma R.A., Falster D.S., Taskinen S. (2012). smatr 3—An R package for estimation and inference about allometric lines: The smatr 3—An R package. Methods Ecol. Evol..

[64] Wickham H. (2016). ggplot2: Elegant Graphics for Data Analysis.

