# Supplementary material for: Paralytic Shellfish Poisoning (PSP) in Mussels from the Eastern Cantabrian Sea: Toxicity, Toxin Profile, and Co-Occurrence with Cyclic Imines
Source: Toxins (Basel). 2021 Oct 27;13(11):761. doi: 10.3390/toxins13110761 (PMC8617803; doi:10.3390/toxins13110761)
Supplement: Supplementary file 1 [file toxins-13-00761-s001.zip › toxins-1408451-supplementary.pdf]

Supplementary Materials: Paralytic Shellfish Poisoning (PSP) in Mussels from the Eastern Cantabrian Sea: Toxicity, Toxin Profile, and Co-Occurrence with Cyclic Imines

Tamara Rodríguez-Cabo, Ángeles Moroño, Fabiola Arévalo, Jorge Correa, Juan Pablo Lamas, Araceli E. Rossignoli and Juan Blanco

Table S1. PSP toxicities estimated (µg STXdiHCl-eq.·kg^{-1}) from the analyses of naturally contaminated mussels from Mendexa, and San Vicente de la Barquera. The sample names are composed of a letter, which indicates the location (M = Mendexa, V = San Vicente de la Barquera), and the month and year when they were collected. Numbers in red correspond to values close but below the LOQ of GTX1,4, which is very high.

		LC-FLD					LC-MS/MS							
**Sample**	**MBA**	**GTX14**	**GTX23**	**STX**	**NEO**	**Total**	**GTX1**	**GTX4**	**GTX2**	**GTX3**	**dcGTX2**	**STX**	**NEO**	**Total**
M-Nov18	1,300	113.6	818.5	51.4	n.d.	983.4	<LOQ	n.d.	523.8	506.7	11	63.3	n.d.	1,104.9
M-Dec18	470	n.d.	129.6	48	n.d.	177.6	<LOQ	<LOQ	88	40.3	<LOQ	53	n.d.	181.3
M-Oct19	810	237.1	102.6	251.2	8	590.9	232.7	65.5	57.5	36.5	n.d.	383.2	125.9	901.5
M-Nov19	430	179.8	93.7	106.3	2	379.8	179.7	33.6	55.1	23.7	n.d.	144.9	53.4	490.5
M-Dec19	480	210.8	178.6	56.6	0	446	162.9	58.4	78.2	84.2	<LOQ	68.6	41.2	493.6
V-Oct19	380	95.9	49	25.5	n.d.	170.4	65.8	n.d.	23.7	13.6	n.d.	27.6	<LOQ	130.6

Table S2. Limits of quantification (LOQs) calculated for each PST. (µg STX di-HCl eq kg^{-1}).

**Toxin**	**LC-FLD**	**LC-MS/MS**
STX	20	0.89
NEO	144	2.08
dcSTX	20	3.93
dcNEO	60	4.68
GTX1	144	3.94
GTX4		3.22
GTX2	20	0.5
GTX3		1.84
C1	20	0.12
C2		0.54
dcGTX2	40	0.86
dcGTX3		1.57
GTX5	2	7.09
GTX6	18	5.65

Table S3. MS/MS fragmentation conditions for paralytic shellfish toxins determination. ESI = Electrospray ionization mode, Q1 = m/z ratio in the first quadrupole, Q3 = m/z ratio in the third quadrupole, DEP(v) = declustering potential, EP(v) = entrance potential, CE(v) = collision energy, and CXP(v) = collision cell exit potential.

Toxin	ESI	Q1	Q3	DEP (v)	EP (v)	CE (v)	CXP(v)
STX*1	POS	300.10	204.10	80	15	31	10
STX*2	POS	300.10	138.00	80	15	37	10
dcSTX*1	POS	257.10	180.10	80	15	29	10
dcSTX*2	POS	257.10	222.10	80	15	21	10
NEO*1	POS	316.10	110.00	80	15	51	10
NEO*2	POS	316.10	164.00	80	15	41	10
dcNEO*1	POS	273.10	180.00	80	15	29	10
dcNEO*2	POS	273.10	162.00	80	15	29	10
GTX1*1	NEG	410.10	367.10	-80	-15	-21	-11
GTX1*2	NEG	410.10	349.10	-80	-15	-29	-11
GTX4*1	NEG	410.10	367.10	-80	-15	-21	-11
GTX4*2	NEG	410.10	349.10	-80	-15	-29	-11
GTX2*1	NEG	394.10	351.10	-80	-15	-22	-11
GTX2*2	NEG	394.10	333.10	-80	-15	-30	-11
GTX3*1	POS	396.102	298.100	-80	-15	27	-11
GTX3*2	NEG	394.10	333.10	-80	-15	-30	-11
dcGTX2*1	NEG	351.10	164.00	-80	-15	-42	-11
dcGTX2*2	NEG	351.10	333.10	-80	-15	-26	-11
dcGTX3*1	NEG	351.10	164.00	-80	-15	-42	-11
dcGTX3*2	NEG	351.10	333.10	-80	-15	-26	-11
C1*1	NEG	474.10	122.00	-80	-15	-38	-11
C1*2	NEG	474.10	351.10	-80	-15	-36	-11
C2*1	NEG	474.10	122.0	-80	-15	-38	-11
C2*2	NEG	474.10	351.1	-80	-15	-36	-11
GTX5*1	POS	380.10	300.10	80	15	19	10
GTX5*2	POS	380.10	282.10	80	15	43	10
GTX6*1	POS	396.10	316.10	80	15	20	10
GTX6*2	POS	396.10	298.10	80	15	31	10

*1 Quantification transition

*2 Confirmation transition

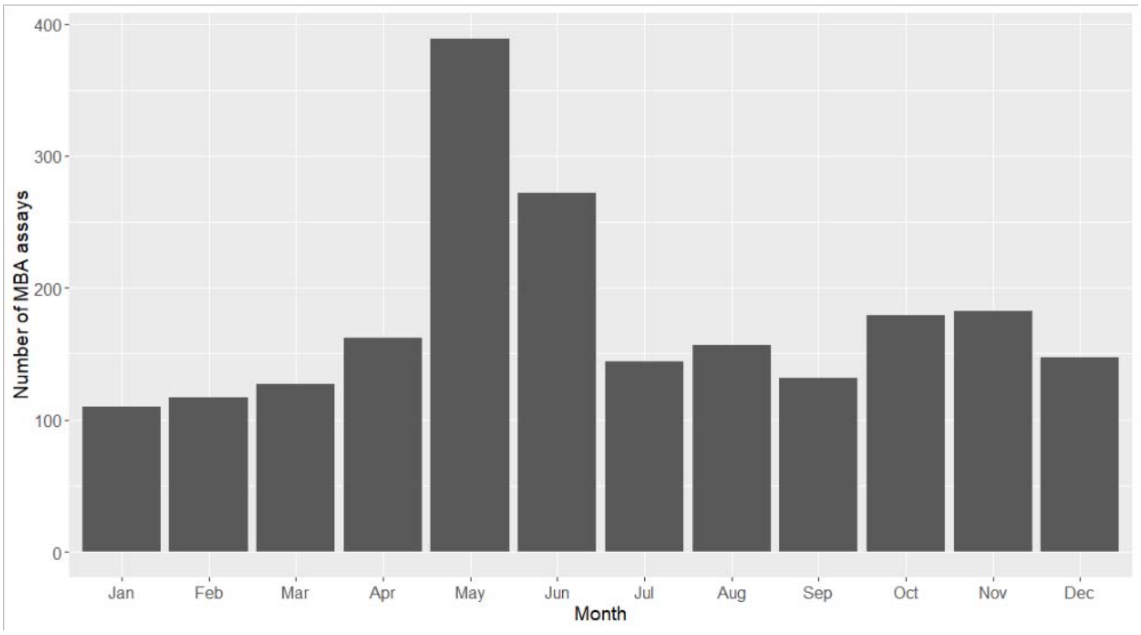

Figure S1. Distribution per month of samples analyzed by MBA by the monitoring system.
